# The diatom test for drowning: an unreported source of diatom contamination

**DOI:** 10.1007/s00414-025-03527-w

**Published:** 2025-06-21

**Authors:** Philippe Lunetta, Johanna Virri, Jan Weckström

**Affiliations:** 1 Department of Biomedicine, Forensic Medicine, University of Turku, Turku, FI-20520 Finland; 2 Forensic Medicine Unit, Finnish Institute for Health and Welfare, P.O. Box 30, Helsinki, FI-00271 Finland; 3 Ecosystems and Environment Research Programme, Faculty of Biological and Environmental Sciences, Helsinki Institute of Sustainability Science (HELSUS), University of Helsinki, Helsinki, FI-00014 Finland

**Keywords:** Drowning, Diatom, Laboratory, Microscope slides, Contamination, Aulacoseira

## Abstract

We describe here a previously unreported source of diatom contamination that may prove relevant in routine diatom analysis in suspected drowning cases. Glass microscope slides utilized in the process, during their manufacture, undergo a washing process, after which, diatomaceous earth (kieselguhr, fossil diatoms) is added to avoid producing stuck slides during the last rinse. The diatomaceous earth can be removed at this point only by means of aggressive cleaning, but the possibility exists that some fossil diatoms will remain on the slides. This potential contamination source explained a recurrent and otherwise puzzling appearance of *Aulacoseira* diatom genus in samples both from definite drowning cases and from non-drowned control cases. Discrimination by expert diatomologists between present-day fresh diatoms and fossil diatoms, together with awareness of the ubiquity of diatoms in nature and of their extensive use in the manufacturing industry, corroborate our view that potential sources of laboratory contamination should not be a rational impediment to performing diatom tests.

## Introduction

The diagnosis of cause of death in corpses retrieved from aquatic settings is complex and is considered among the most difficult in autopsy practice. Even in definite drowning, macroscopical changes as well as histological and laboratory findings show great variability [1–4]. A wide range of potential indicators for drowning, as lung surfactant and aquaporins, have been investigated [1, 5, 6], but the low cost-efficiency seems to limit their wide application in caseworks. The diatom test is used as supportive evidence for the diagnosis of drowning in certain countries [1, 7–11], but divergent opinions exist on its reliability [9, 10]. A crucial issue is the occurrence of false-positive cases i.e. diatoms detectable in individuals who did not die from drowning. Because diatoms are ubiquitous in water, soil, and air and are widely used in the manufacturing industry, false-positive cases can be related to in vivo penetration into organs (gastro-enteric absorption, inhalation) or post-mortem passive diffusion during submersion [1, 9–11]. False positives may also result from autopsy and laboratory contamination of organ samples [1, 9–12]. A strict protocol is thus necessary to avoid contamination during the entire sequence of diatom preparation, from tissue sampling at autopsy to sample mounting onto the slides. In this paper, we illustrate a previously unreported source of diatom contamination that can, if unrecognized, hamper the interpretation of diatom analysis in suspected drowning cases.

## Materials and methods

**Fig. 1a, b:**
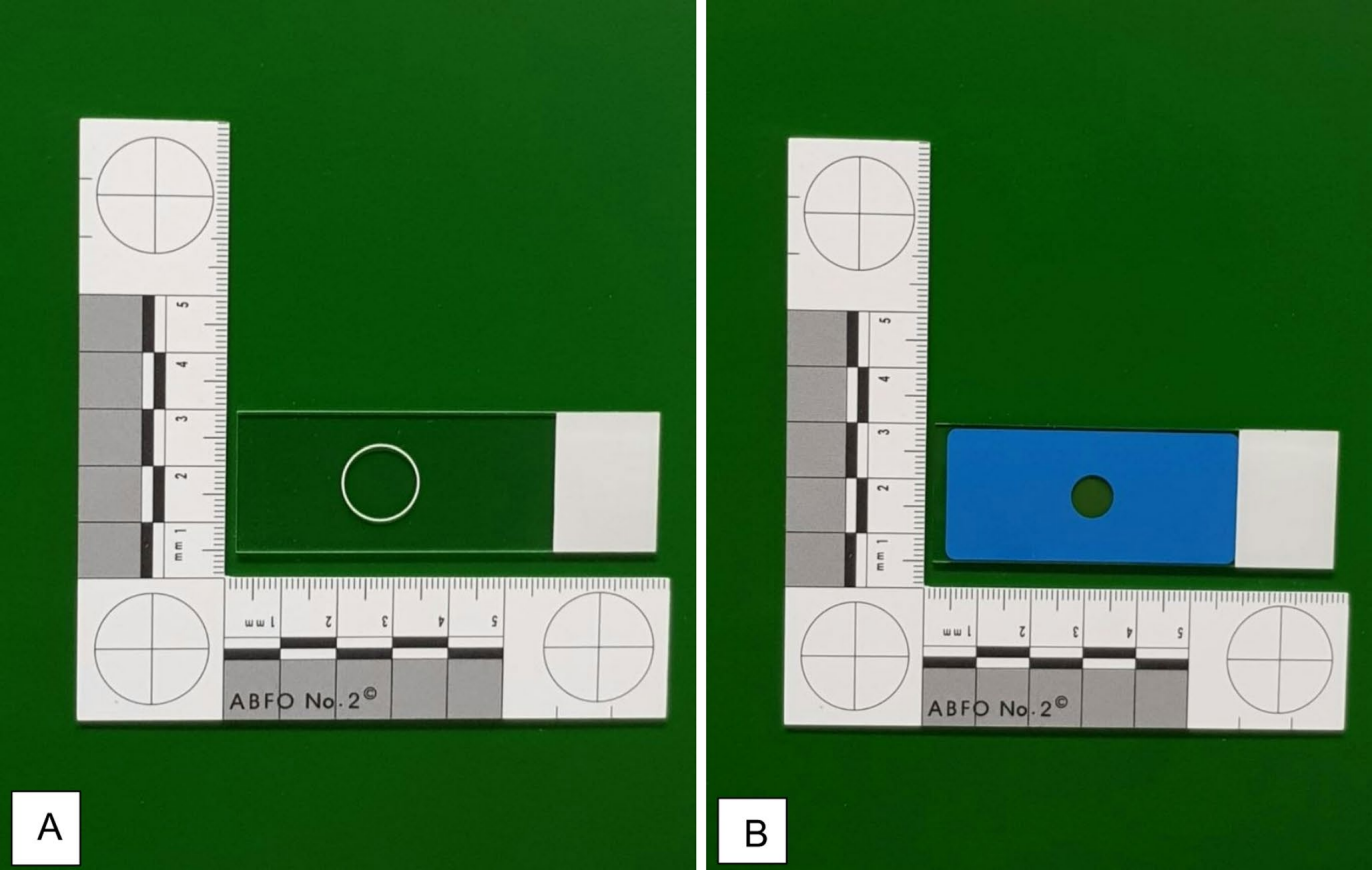
Non-adhesive microscope glass slides: (**a**) Epoxy-type slide, (**b**) PTFE-type slide

In Finland, approximately 120 to 160 medicolegal autopsies, that include a diatom test for the diagnosis of drowning, are performed annually at the Forensic Medicine Unit of the National Institute for Health and Welfare (NIHW) on corpses found in water. In such cases, laboratory personnel at the NIHW Forensic Medicine Unit process all relevant samples taken at autopsy and prepare glass microscope slides for taxonomic analysis of diatoms by an expert diatomologist (one of the authors, JW). In addition to the water samples from the putative drowning site, the most common samples analyzed are from victims’ lung, brain, liver, kidney, and, in some cases, bone-marrow tissues. Samples are prepared by the standard acid digestion method, as previously described, using nitric acid and hydrogen peroxide [12]. To minimize the area to be studied by light microscopy (LM), following tissue digestion and centrifugation, a drop (or drops) of each sample are deposited on a demarcated area of a non-adhesive microscope slide. Slides (Superfrost, Gerhard Menzel GmbH, Thermo Scientific) originally used for those analyses were non-adhesive, with a hand-made circle added at the laboratory by permanent marker. Those slides were first replaced by non-adhesive epoxy slides (Diagnostic microscope slide, Gerhard Menzel GmbH, Thermo Scientific) with a white ring 13 mm in diameter, and then, more recently, by non-adhesive PTFE diagnostic slides (Diagnostic microscope slide, Gerhard Menzel GmbH, Thermo Scientific) (Fig. 1a, b). The microscopy analyses are carried out with an Olympus BX40 microscope with phase-contrast at 400-1000x magnification. The laboratory personnel assisted by the expert diatomologist perform regular checks to exclude contamination issues involving reagents and other equipment for preparation of these diatom samples.

## Results and discussion

**Fig. 2a, b:**
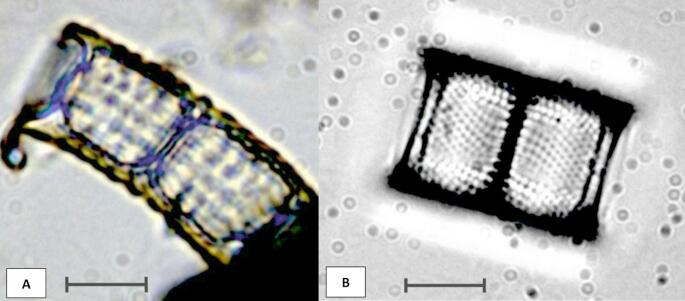
Remains of *Aulacoseira* species: (**a**) contaminating *Aulacoseira cf. temperei* (1000x); (**b**) common freshwater *Aulacoseira ambigua* (1000x) (scale bar = 10 μm)

Of 1440 consecutive non-adhesive microscope epoxy and PTFE slides analyzed for diatoms, we detected in at least 1074 (74.6%) slides the remains of contaminating *Aulacoseira* spp. (Thwaites 1848) [13, 14] (Fig. 2a, b), a fossil diatom species occurring in diatomaceous earth (kieselguhr), dating back in time some 10 to 12 million years. The number of these diatom valves per slide ranged from 0 to 69, with a mean occurrence of 8 diatom valves per contaminated slide.

The detection of such unusual valves of the diatom genus *Aulacoseira*, not only in drowning cases, but also in our control samples from non-drowned cases, led us to seek a possible contamination source. We systematically tested, in different combinations, the environment, reagents, and other equipment used for preparation of diatom samples, namely laboratory glassware, distilled water, cellulose wadding, coverslips, mounting medium (Naphrax^®^), and microscope glass slides either as extracted from intact packages or exposed to air within a fume cupboard.

The laboratory assessment of the origin of the *Aulacoseira* spp. diatom contamination allowed us to exclude all of these environmental sources of contamination and to identify the contamination source as being the non-adhesive epoxy and PTFE microscope slides currently used in our laboratory for microscopic diatom analysis (Fig. 1a, b). To further narrow the origin of the *Aulacoseira* spp., we requested additional information from the microscope glass slide manufacturer, namely whether diatomaceous earth (kieselguhr) is currently used during the slide manufacturing process. A manufacturer’s representative (D. Fricke, Thermo Fisher Scientific, personal communication) confirmed that no diatomaceous earth is present in the ink used to demarcate the circular area of the slide. Diatomaceous earth is, however, used during the cleaning process of non-adhesion glass to avoid producing stuck slides. The washing process sees diatomaceous earth added to the glass during the last rinsing step. The slides are dried and the diatomaceous earth can be removed at this point only by means of very aggressive cleaning, but the possibility still exists that some fossil diatoms will remain on the slides. Since many diatomaceous earth deposits are monotaxonic and comprised of variable fossil *Aulacoseira* species [15, 16] it is likely that this genus will be found on slides, although also other genera, ones present in diatomaceous earth such as *Stephanodiscus* and *Cyclotella*, might eventually be found [17]. The contaminating fossil diatoms in this study resembled *Aulacoseira temperei* [15], but this could not yet be confirmed, because the manufacturer provided no information on the actual origin of the diatomaceous earth used during slide manufacture. Of note is that the manufacture of adhesion slides requires no diatomaceous earth, because the chemicals used for adhesion already ensure that the slides remain separated.

Routine taxonomic diatom analysis of lungs and other organs by LM often reveals valves of present-day *Aulacoseira* spp., especially in drowning cases occurring in fresh waters (Fig. 2b). This is not surprising, as *Aulacoseira* is one of the most common freshwater diatom taxa [18]. As some of the present-day *Aulacoseira* species may resemble the *Aulacoseira* spp. material from the diatomaceous earth, taxonomic and discrimination problems may arise between water-borne *Aulacoseira* and those present in the diatomaceous earth stuck onto the glass surface. However, the overall structure of the fossil *Aulacoseira* cf. *temperei* is more robust than that of the present-day diatom species (Fig. 2a, b). In addition to other structural differences [15], its cell wall is usually notably thicker than the cell wall of the present-day *Aulacoseira* species [15]. Therefore, this contamination issue with potential false-positive results, if properly acknowledged, can relatively easily be recognized and handled by an expert diatomologist.

## Conclusion

In performing the diatom test for the diagnosis of drowning, standardized methods for diatom analysis, including a search for and an identification of contamination sources and exclusion of any potential “contaminating” diatoms are crucial. In most lung samples with a high concentration of different species of diatoms, the eventual presence of isolated contaminating diatoms does not interfere with the diatom test’s overall results and interpretation. However, in internal organs where, even in actual drowning cases, diatom valves appear sporadically or in very limited quantities, a few contaminating diatoms, such as *Aulacoseira* spp. in our case, may pose significant diagnostic problems. For these reasons, during LM diatom analysis in cases of drowning, adhesive microscopy glass slides requiring no use of diatomaceous earth during their manufacture should be preferred to non-adhesive microscopy glass slides. Even for use of non-adhesive slides, an expert diatomologist should, however, have no problems in separating fossil contamination taxa from freshwater taxa, because modern and fossil species show clear structural differences. Thus, in diatom testing, if appropriately dealt with, possible laboratory contamination should not prove a rational impediment.
